# Glycosylation in lymphoma: Biology and glycotherapy

**DOI:** 10.1111/pin.12834

**Published:** 2019-07-17

**Authors:** Osamu Suzuki

**Affiliations:** ^1^ Department of Diagnostic Pathology, School of Medicine Fukushima Medical University Fukushima Japan

**Keywords:** cell adhesion, cell death, N‐glycans, sialic acid

## Abstract

Research using mouse lymphoma cell lines has resulted in many reports of glycosylation being a key regulator for the distant metastasis of mouse lymphoma cells in animal models. In contrast, there are only a few reports of experiments examining human lymphoma cell metastasis. The glycosylation pattern in human lymphoma shows that loss of *Phaseolus vulgaris* leukoagglutinating lectin (L‐PHA) reactive oligosaccharides, and sialylation of L‐PHA reactive oligosaccharides, are closely associated with a worse prognosis for diffuse large B cell lymphoma (DLBCL) patients. Sialic acid is related to cell adhesion to the extracellular matrix and metastasis of HBL‐8 Burkitt lymphoma cells in a severe combined immunodeficiency (SCID) mouse animal model. In HBL‐8 clones, differential cell surface sialylation was due to different expression levels of UDP‐GlcNAc 2‐epimerase (GNE). Knockdown of beta‐galactoside alpha‐2,6‐sialyltransferase (ST6Gal1) resulted in enhanced lymphoma cell adhesion to galectin‐1 in anaplastic large cell lymphoma cell line, H‐ALCL. A fluorinated sialic acid analogue was shown to be useful for inhibiting sialyltransferase and may provide a new glycoengineering strategy for desialylation, as well as inhibiting invasion and metastasis and inducing cell death in lymphoma cell lines. This paper discusses glycosylation and sialylation in human lymphoma, and several glycoengineering therapeutic strategies for lymphoma.

## BACKGROUND

**Figure 1:**
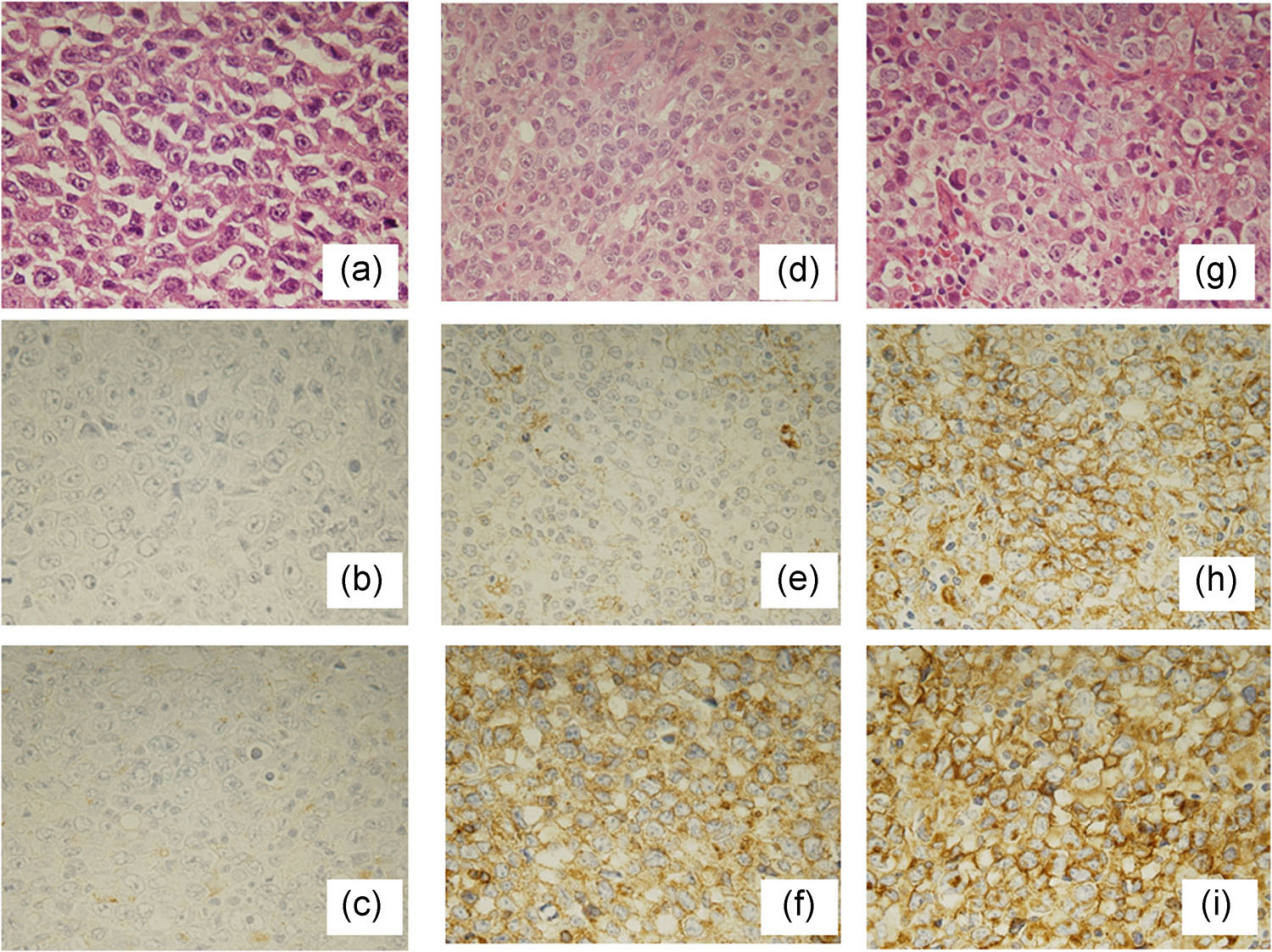
Representative findings of *Phaseolus vulgaris* leukoagglutinating lectin (L‐PHA) lectin histochemistry. (**a**) H.E staining of loss of L‐PHA oligosaccharides case. (**b**) L‐PHA staining without neuraminidase pretreatment of loss of L‐PHA oligosaccharides case. Tumor cells are negative for L‐PHA. (**c**) L‐PHA staining with neuraminidase pretreatment of loss of L‐PHA oligosaccharides case. Tumor cells are negative for L‐PHA. (**d**) H.E staining of sialylated L‐PHA oligosaccharides case. (**e**) L‐PHA staining without neuraminidase pretreatment of sialylated L‐PHA oligosaccharides case. Tumor cells are negative for L‐PHA. (**f**) L‐PHA staining with neuraminidase pretreatment of sialylated L‐PHA oligosaccharides case. Tumor cells show cell surface staining of L‐PHA. (**g**) H.E staining of non‐sialylated L‐PHA oligosaccharides case. (**h**) L‐PHA staining without neuraminidase pretreatment of non‐sialylated L‐PHA oligosaccharides case. Tumor cells show cell surface staining of L‐PHA. (**i**) L‐PHA staining with neuraminidase pretreatment of non‐sialylated L‐PHA oligosaccharides case. Tumor cells show cell surface staining of L‐PHA. The loss of L‐PHA oligosaccharides case and sialylated L‐PHA oligosaccharides case are non‐germinal center B cell like type, and non‐sialylated L‐PHA oligosaccharides case is a germinal center B cell like type. But statistical analysis of correlation between the L‐PHA staining pattern and non‐germinal center B (GCB) classification has not yet been published.

Glycosylation plays a significant role in the cell biology of many malignant tumors. For example, the concentration of sialic acid has been reported to be elevated in the serum of patients with malignant lymphoma,^1^ suggesting that sialic acid may be associated with tumor cell aggressiveness in lymphoma. In many cancers, glycosylation is closely related to the patient's prognosis: *Phaseolus vulgaris* leukoagglutinating lectin (L‐PHA) reactive oligosaccharides are reported to be associated with patients’ prognosis in non‐small cell lung cancer,^2^ and alpha 2,6‐sialic acid and ST6Gal1 are upregulated in hepatoma and colon cancers.^3^ Alpha 2,6‐sialic acid may play a significant role in carcinogenesis in hepatoma or colon cancer. Sialic acids are linked to some galactose termini of L‐PHA lectin reactive oligosaccharides. Sialylation of the cell surface of a mouse lymphoma cell line is associated with a high metastatic rate,^5^ and in human malignant lymphoma, sialylation of L‐PHA reactive oligosaccharides appears to be closely associated with a worse prognosis and an advanced clinical stage.^6^, ^7^ Loss of L‐PHA reactive oligosaccharides and their alpha 2,6‐sialylation is related to a worse prognosis for patients with diffuse large B cell lymphoma (DLBCL).^7^ The staining pattern of L‐PHA in combination with neuraminidase treatment is shown in Fig. 1. Lectin blotting using L‐PHA lectins showed that several glycoproteins are correlated to those observed by L‐PHA lectin histochemical staining of paraffin embedded tissue sections.^6^ Variations in DLBCL were classified by Hans,^8^ of which the non‐germinal center B (GCB)‐like type and GCB‐like type are correlated to our classification of L‐PHA lectins identified by histochemical staining of DLBCL tissue sections as shown in Fig. 1. Patients with lymphoma categorized as non‐GCB‐like show a worse prognosis, similar to sialylated L‐PHA reactive cases, whereas GCB‐like type cases show a favorable prognosis, similar to non‐sialylated L‐PHA reactive cases. The non‐GCB/GCB‐like type classifications may be correlated to the sialylation pattern of L‐PHA reactive oligosaccharides observed by L‐PHA lectin histochemical staining. But statistical analysis has not yet been published.

**Figure 2:**
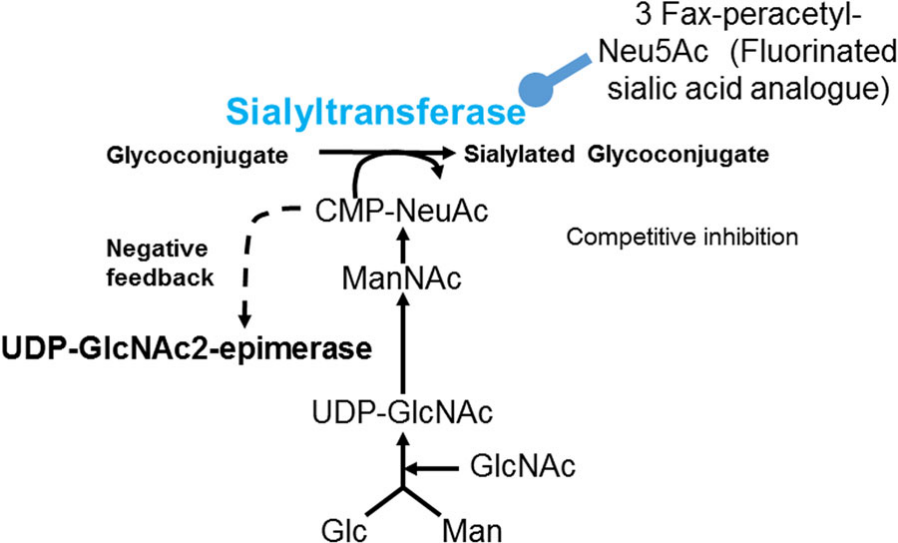
Biosynthetic pathway of sialic acid. Schematic representation of the sialic acid biosynthetic pathway. UDP‐GlcNAc2‐epimerase (GNE) catalyzes the rate‐limiting step in the biosynthesis of sialic acid. The activity of UDP‐GlcNAc2‐epimerase is negatively regulated by CMP‐NeuNAc. NeuAc, N‐acetyl neuraminic acid; CMP‐NeuAc, cytidine monophosphate‐N‐acetyl neuraminic acid; ManNAc, N‐acetyl‐D‐mannosamine; UDP‐GlcNAc, uridine diphosphate‐N‐acetyl‐D‐glucosamine; GlcNAc, N‐acetyl‐D‐glucosamine; Glc, D‐glucose; Man, D‐mannose. This figure is from reference^10^, with slight modification. GNE is a key regulator for biosynthesis of sialic acid. Cell surface sialylation is medicated by sialyltransferase, for example, ST6Gal1, in combination with GNE.

Altevogt *et al.* used the mouse lymphoma cloned cell lines Esb and Eb and reported that sialylation is closely associated with distant metastasis in a mouse animal model. The Esb cell line has sialylated glycans and showed a higher metastatic phenotype than the Eb clone, which has non‐sialylated glycans. Two different clones are available for the Burkitt lymphoma cell line HBL‐8. Abe *et al.* reported that the 3G3 clone of HBL‐8 has a highly sialylated cell surface and is highly metastatic whereas the 3D2 clone of HBL‐8 has a low level of cell surface sialylation and is poorly metastatic in a severe combined immunodeficiency (SCID) mouse animal model.^9^ This was the first report that differential cell surface sialylation is closely associated with distant metastasis in an animal model using a human lymphoma cell line, as well as of a clear difference in sialic acid content between the 3G3 and 3D2 HBL‐8 clones. Furthermore, UDP‐GlcNAc 2‐epimerase (GNE) is a key enzyme for sialic acid biosynthesis as shown in Fig. 2, however, reverse transcription polymerase chain reaction (RT‐PCR) analysis indicates that GNE messenger RNA (mRNA) is expressed in the 3G3 clone but is not expressed in the 3D2 clone,^10^ suggesting that GNE is a key regulator for sialic acid biosynthesis in HBL‐8 clones and the observed differences in sialic acid content are due to differences in the mRNA expression of GNE. Sialic acid precursor complementation assay also revealed the loss of activity of GNE in the poorly sialylated 3D2 clone. In addition, there is a significant difference in cell adhesion to extracellular matrix and in the cell growth rate of the two clones, with 3G3 exhibiting lower cell adhesion to extracellular matrix and higher growth rate compared to 3D2. The addition of N‐acetyl‐mannosamine (ManNAc), a precursor of sialic acid, to the 3D2 culture medium resulted in cell surface resialylation, detection of the sialic acid‐specific lectin from *Limax flavus,* and enhanced cell growth. Resialylation by ManNAc inhibits ceramide‐induced cell death and inhibits necrosis and death due to lactate dehydrogenase (LDH) release from the cytoplasm caused by membrane permeability, suggesting that resialylation may be closely associated with enhanced HBL‐8 cell growth or the inhibition of cell death. RT‐PCR and flow cytometry using small interfering RNA (siRNA) for GNE showed that the knockdown of GNE in the 3G3 clone of HBL‐8 reduced GNE mRNA in real time and decreased cell surface sialylation of the sialic acid‐specific lectin from *Maachia amurensis*.^11^ In addition, reduced cell surface sialylation on the 3G3 clone of HBL‐8 by knockdown of GNE reduced the cell growth rate and increased sensitivity to ceramide‐induced cell death. Taken together, these previous results suggest that our research on L‐PHA reactive oligosaccharides and sialic acid, both on lymphoma and using human lymphoma cell lines, may be closely associated with the clinical outcome of patients with lymphoma and on lymphoma cell biology.

## L‐PHA REACTIVE OLIGOSACCHARIDES IN DLBCL

L‐PHA reactive oligosaccharides in lymphoma modulate cell adhesion to extracellular matrix^12^ via a mechanism possibly related to extracellular matrix receptor, integrins, or to Rho family activation. Integrins and Rho family proteins are involved in cytoskeleton reorganization and tumor cell motility. We speculate that N‐glycans on integrins control integrin activation, resulting in the regulation of Rho family proteins and cell motility. N‐glycans, including L‐PHA reactive oligosaccharides, may regulate lymphoma cell adhesion to extracellular matrix, especially in relation to integrins. Tunicamycin, a potent inhibitor of N‐glycosylation, appears to enhance HBL‐8 cell adhesion to fibronectin. Several studies have suggested that both glycosylation and sialic acid in integrins regulate integrin activity during tumor cell adhesion to extracellular matrix.^13^, ^14^, ^15^, ^16^ Therefore, N‐glycans and sialic acid may be significant regulators for tumor cell adhesion to extracellular matrix, perhaps by altering the molecular conformation of integrin by steric hindrance, thereby modulating the function of integrin. Sialic acid is negatively charged and this charge affects the interaction between sialic acid and extracellular matrix. The weakening of cell adhesion to extracellular matrix by sialic acid on the cell surface may facilitate more invasive phenotypes of lymphoma cells, leading to highly invasive activity or increased metastatic rate. LAMP‐1, which exhibits a heterogeneous pattern of N‐glycosylation (L‐PHA reactive oligosaccharides), is involved in autophagy.^17^ Since autophagy may regulate drug resistance in cancer cells,^18^ we will in future clarify the significance of LAMP‐1 in autophagy and in the chemoresistance of malignant lymphoma cells.

N‐glycans, including L‐PHA reactive oligosaccharides of IgM, regulate cell growth induced by the CD40‐CD40L system in DLCBL.^19^ As L‐PHA lectin recognizes complex type N‐linked oligosaccharides, complex type N‐glycans may be linked to IgM. Swainsonine treatment inhibits the N‐glycosylation of IgM, enhances cell rescue effects by the CD40‐CD40L system, and enhances the expression of bcl‐2, an inhibitor of apoptosis. IgM signaling works in conjunction with the CD40‐CD40L system in B cells.^20^ It was previously reported that N‐glycans on immunoglobulin in follicular lymphoma may regulate cell growth by interacting with dendritic cell‐specific intracellular adhesion molecule 3‐grabbing non‐integrin (DC‐SIGN) lectin.^21^ It was also reported that galectin‐1 expressed in stromal cells induces IgM signaling by interacting with the glycans of IgM.^22^ These studies suggest that interactions between N‐glycans and lectins in hematopoietic cells may help regulate cell growth and/or adhesion. The PI3K‐Akt system may be involved in numerous biological phenomena, but details of the biological mechanisms remain unclear and further study using lymphoma cell lines is needed to clarify the PI3K‐Akt system. Nonetheless, we speculate that the Rho family member Cdc42 may be associated with lymphoma cell motility and invasion and thus Cdc42 may be involved in lymphoma cell adhesion to galectin‐3 and in lymphoma cell motility involving galectin‐3. Galectin‐3 regulates lymphoma cell adhesion and invasion through signaling, thereby modulating lymphoma cell metastasis. Recent research has shown that IgM signaling helps drive lymphoma development.^23^ IgM signaling stimulated multidirectional signals, such as CD79a, CD79b, Lck/Yes novel tyrosine kinase (LYN), spleen tyrosine kinase (SYK), Bruton's kinase (BTK), caspase recruitment domain‐containing protein 11 **(**CARD11), and PI3K‐Akt. Akt signaling is bidirectional, with one activity aiding survival and the other aiding apoptosis.^23^ Therefore, IgM ligation by anti‐IgM antibody may induce cell growth or cell death, depending on the balance between prosurvival and proapoptotic signals. These bidirectional signals in lymphoma may be similarly be regulated by a balance between prosurvival and proapoptotic signals.

## SIALYLATION IN THE ADHESION AND INVASION OF LYMPHOMA CELLS

**Figure 3:**
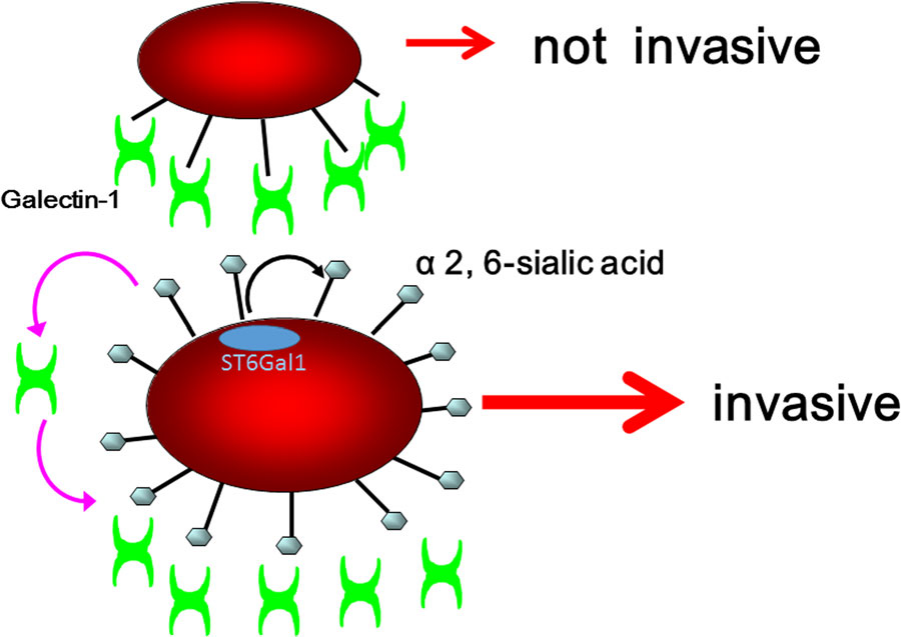
ST6Gal 1 synthesizes α2,6‐sialylation to more invasive phenotype. Desialylation enhances lymphoma cell adhesion and inhibits cell invasiveness. On the other hand, sialylation of glycans on the cell surface enhances cell invasiveness. Sialylation may facilitate lymphoma cell invasiveness by moderately weakening cell adhesion to galectins found in the extracellular matrix.

**Figure 4:**
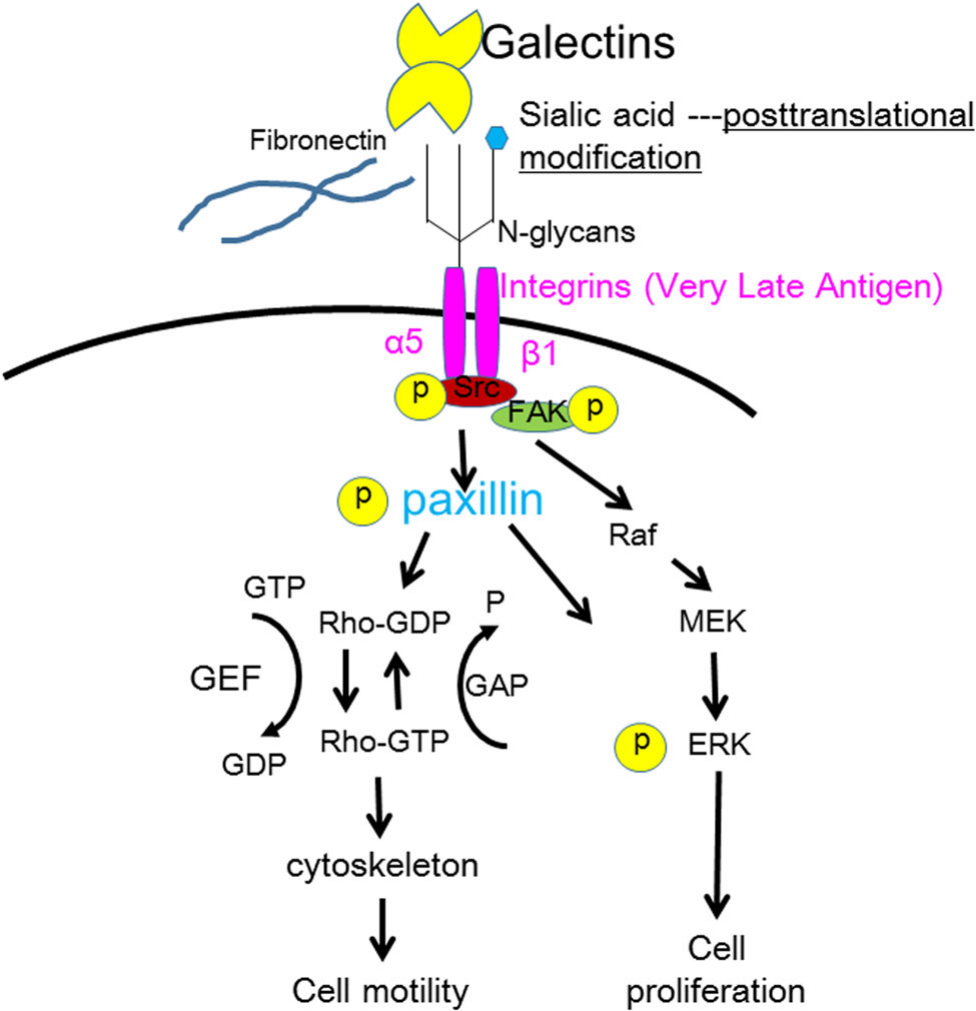
Sialic acid and integrin signaling. Posttranslational modification of glycans on integrins by sialylation regulates integrin signaling in lymphoma, increasing the invasiveness of lymphoma cells. Integrins are associated with paxillin and Rho family proteins, which modulate cell motility. P, phosphorylation; GTP, guanosine triphosphate; GDP, guanosine diphosphate; GEF, guanine nucleotide exchange factor; GAP, Rho GTPase‐activating protein; src, proto‐oncogene tyrosine‐protein kinase src; FAK, focal adhesion kinase; Raf, redox factor‐1; MEK, mitogen activated protein kinase; ERK, extracellular signal‐regulated kinase.

**Figure 5:**
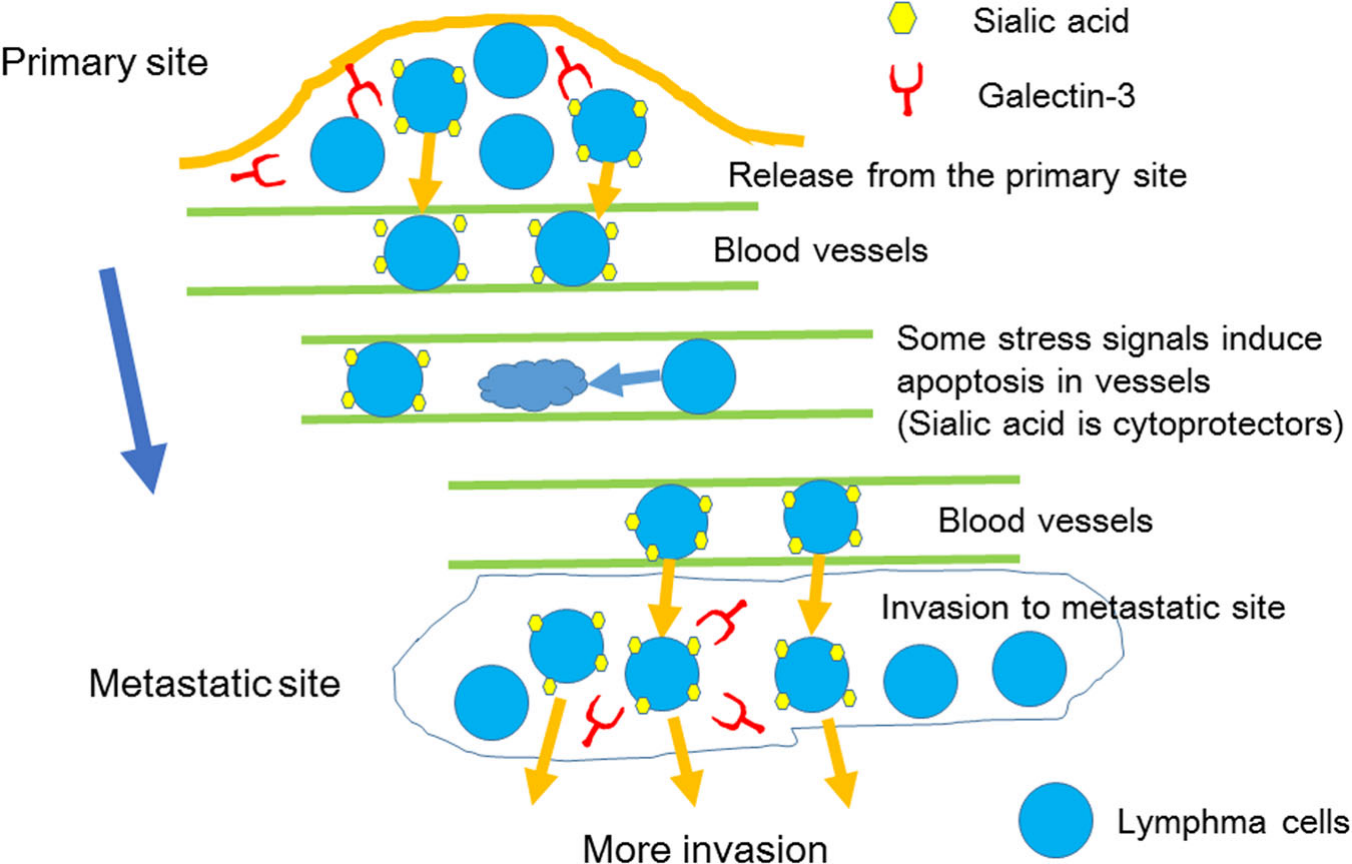
Schematic representation of lymphoma cell metastasis. Cell surface sialylation may facilitate the release of lymphoma cells from the primary tumor site into the bloodstream, and extravasation of lymphoma cells to the metastatic site and more invasion. Stress may induce the apoptosis of lymphoma cells in vessels. However, sialylation protects lymphoma cells from stress signals and may rescue lymphoma cells from apoptosis. Sialic acid is a regulator of lymphoma cell invasion and metastasis.

In malignant lymphoma, cell adhesion to or invasion of the extracellular matrix are significantly related to lymphoma cell metastasis.^10^ Using the anaplastic large cell lymphoma cell line H‐ALCL, we analyzed whether cell adhesion to galectins or extracellular matrix is regulated by ST6Gal1.^24^, ^25^ Knockdown of ST6Gal1 resulted in enhanced cell adhesion to galectin‐1, galectin‐8, and laminin. Galectin is one of the lectins which react to oligosaccharides and galectin is a soluble form (called S‐type) lectin. Among galectins, galectin‐3 has a homologue to bcl‐2 family protein and galectin‐3 reacts to bcl‐2 for control of cell apoptosis. Galectin is found in the extracellular matrix and laminin is a component of the basement membrane of vessels. Therefore, ST6Gal 1 may regulate cell adhesion to the extracellular matrix, including to galectins, and induce extravasation through laminin in the surrounding vessels of distant organs. Sialylation by ST6Gal1 may facilitate cell invasiveness of the extracellular matrix by weakening cell adhesion to the extracellular matrix, as shown in Fig. 3. Desialylation of the H‐ALCL cell surface glycans by neuraminidase treatment markedly enhanced cell adhesion to galectin‐1 and inhibited cell invasion by galectin‐1. The marked enhancement of lymphoma cell adhesion to galectin‐1 led to low motility involving galectins due to marked adhesion to galectin‐1. Therefore, moderately weakened cell adhesion resulting from cell surface sialylation may be required to enhance cell motility. Furthermore, the Rho inhibitor C3‐transferase inhibits the invasion H‐ALCL cells by galectin^24^ and thus the Rho family may help regulate lymphoma cell invasion by galectins. Furthermore, cell adhesion to or invasion by galectin‐1 was inhibited by pretreatment with RGD peptide, a specific inhibitor of alpha5beta1‐integrins. Therefore, cell adhesion to or invasion by galectin‐1 is mediated by alpha5beta1‐integrin and may be regulated by the glycosylation or sialylation of integrins. In recent research N‐glycosylation of integrin is a key regulator for cell adhesion.^26^ Integrins interact with Rho proteins and thus sialylation may modulate integrin activity, leading to control of Rho family function. Furthermore, cytochalasin B inhibits lymphoma cell invasion, suggesting that the cytoskeleton may be involved in lymphoma cell invasion.^24^ Paxillin is known to regulate and reorganize the cytoskeleton, enhancing the adhesion and motility of cells. Therefore, altered sialylation might regulate cell adhesion and the phosphorylation of paxillin, as shown in Fig. 4. Sialylation may lead to a more invasive phenotype of lymphoma cells through integrin‐Rho‐paxillin collaboration. In our opinion, cell surface sialylation may facilitate the release of lymphoma cells from the primary tumor site to the bloodstream, resulting in the extravasation of lymphoma cells to the metastatic site and more invasion, as shown in Fig. 5. Stress within the vessels may induce the apoptosis of lymphoma cells, whereas sialylation is known to protect lymphoma cells from stress signals^11^ and may rescue lymphoma cells from apoptosis. We speculate that sialylation of cell surface glycoproteins may regulate cell adhesion in lymphoma, and desialylation by neuraminidase or knockdown of ST6Gal1 may enhance cell adhesion to galectin or extracellular matrix, thereby inhibiting the invasiveness or metastasis of lymphoma cells and providing a potential strategy for lymphoma glycotherapy by desialylation.

## MODULATED ADHESION OR ONCOLYSIS BY DESIALYLATION IN LYMPHOMA GLYCOTHERAPY

**Figure 6:**
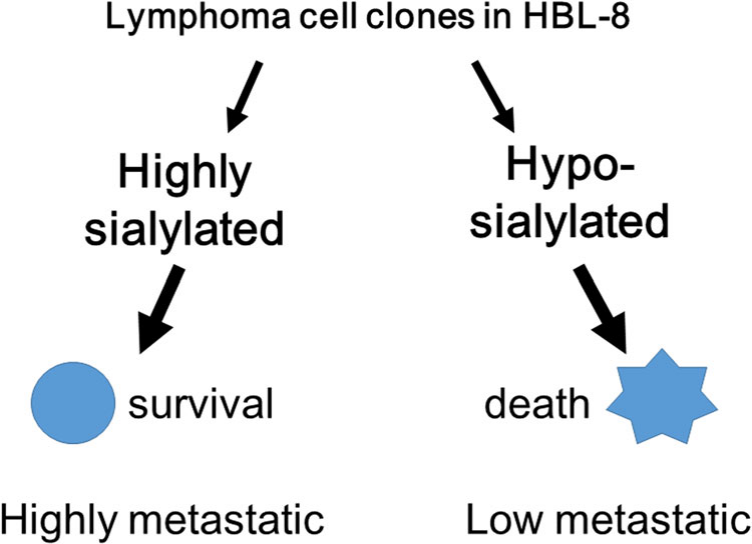
Sialylation in lymphoma cell metastasis. In HBL‐8 clones, a clone with highly sialylated glycans escapes cell death induced by stress or anticancer drugs, leading to a high metastatic rate compared to a clone which has poorly sialylated glycans.

In our opinion, since glycosylation appears to be closely associated with tumor cell biology in human malignant lymphoma, the modulation of cell surface glycosylation by enzymatic (neuraminidase treatment or glycosylation inhibitors) or genetic (knockdown by siRNA) approaches could be useful for lymphoma therapy. We have continued to clarify detailed mechanisms in glycobiology pertaining to lymphoma cell adhesion, invasion, and apoptosis. Neuraminidase treatment or knockdown of ST6Gal1 enhances cell adhesion to galectin‐1, ‐3, ‐8, and laminin, and inhibits lymphoma cell invasion.^24^, ^25^ Marked enhancement of cell adhesion to galectins or extracellular matrix may inhibit the motility or invasion of lymphoma cells into the extracellular space. The marked enhanced cell adhesion by neuraminidase treatment may lead to difficulty for cell movement in lymphoma cells. In our previous research invasion to galectin‐1 is inhibited by neuraminidase treatment in lymphoma cells.^24^ Therefore, neuraminidase treatment or knockdown of ST6Gal1, which enhance cell adhesion to the extracellular matrix, may be candidates as tumor therapies by inhibiting lymphoma cell invasion and metastasis. Desialylation of cloned cancer cells induces Fas‐mediated apoptosis and drug‐induced apoptosis.^27^, ^28^ Desialylation of lymphoma cell surfaces by neuraminidase treatment is associated with enhanced Fas‐ or drug‐induced apoptosis.^29^, ^30^, ^31^ In our research, etoposide‐induced apoptosis appeared to be enhanced by neuraminidase treatment,^32^ and there is a possibility that drug resistance may be regulated by cell surface sialylation in the HBL‐2 cell line. This enhanced apoptotic effect by neuraminidase treatment is dependent on caspase‐3, ‐8, and ‐9. We speculate that since removal of cell surface sialic acid by knockdown of GNE induces HBL‐8 cell death, oncolysis of lymphoma cells may be induced by neuraminidase treatment or knockdown of ST6Gal1 or GNE. Knockdown of ST6Gal1 reduces alpha 2,6‐sialylation of the terminus of N‐glycosylation which is a beta‐galactose. On the other hand, knockdown of GNE reduces the total sialic acid content. There is a clear difference in the desialylation pattern between knockdown of ST6Gal1 and of GNE, but this strategy may provide the same effect on the desialylation of lymphoma cells, resulting in the induction of cell death. Desialylation could thus induce lymphoma cell oncolysis and may provide a new therapeutic strategy, although the detailed mechanisms are still being investigated. As described above, the highly sialylated clone 3G3 shows a high metastatic rate and the poorly sialylated clone 3D2 shows a low metastatic rate in a SCID mouse animal model, as shown in Fig. 6. Knockdown of GNE in HBL‐8 3G3 cells appears to inhibit cell growth and enhance cell death caused by ceramide, an inducer of apoptosis or cell death.^11^ The survival of tumor cells from stress such as hypoxia, oxidative stress, or apoptosis may be related to increased metastatic tumors, and thus the high survival rate of 3G3 clone cells following sialylation may be associated with high metastasis in a SCID animal model. Enhanced cell death in the poorly sialyated 3D2 clone is associated with a low metastatic rate of lymphoma cells in vivo. Desialylation by knockdown of GNE may help inhibit cell growth and induce spontaneous cell death, and lead to a low metastatic rate in a SCID mouse animal model. Therefore, in addition to ST6Gal1, GNE is also a candidate target for lymphoma therapy, especially for the prevention of metastasis. Inhibition of ST6Gal1 or GNE activity in lymphoma cells may cause apoptosis, thereby reducing tumor mass or metastatic nodules and enhancing the effects of anticancer drugs. Future strategies involving desialylation by knockdown of ST6Gal or GNE, or the use of synthetic drugs to inhibit ST6Gal or GNE, may help inhibit cell growth and induce cell death.

## GALECTIN AND CD30 SIGNALING IN ANAPLASTIC LARGE CELL LYMPHOMA

**Figure 7:**
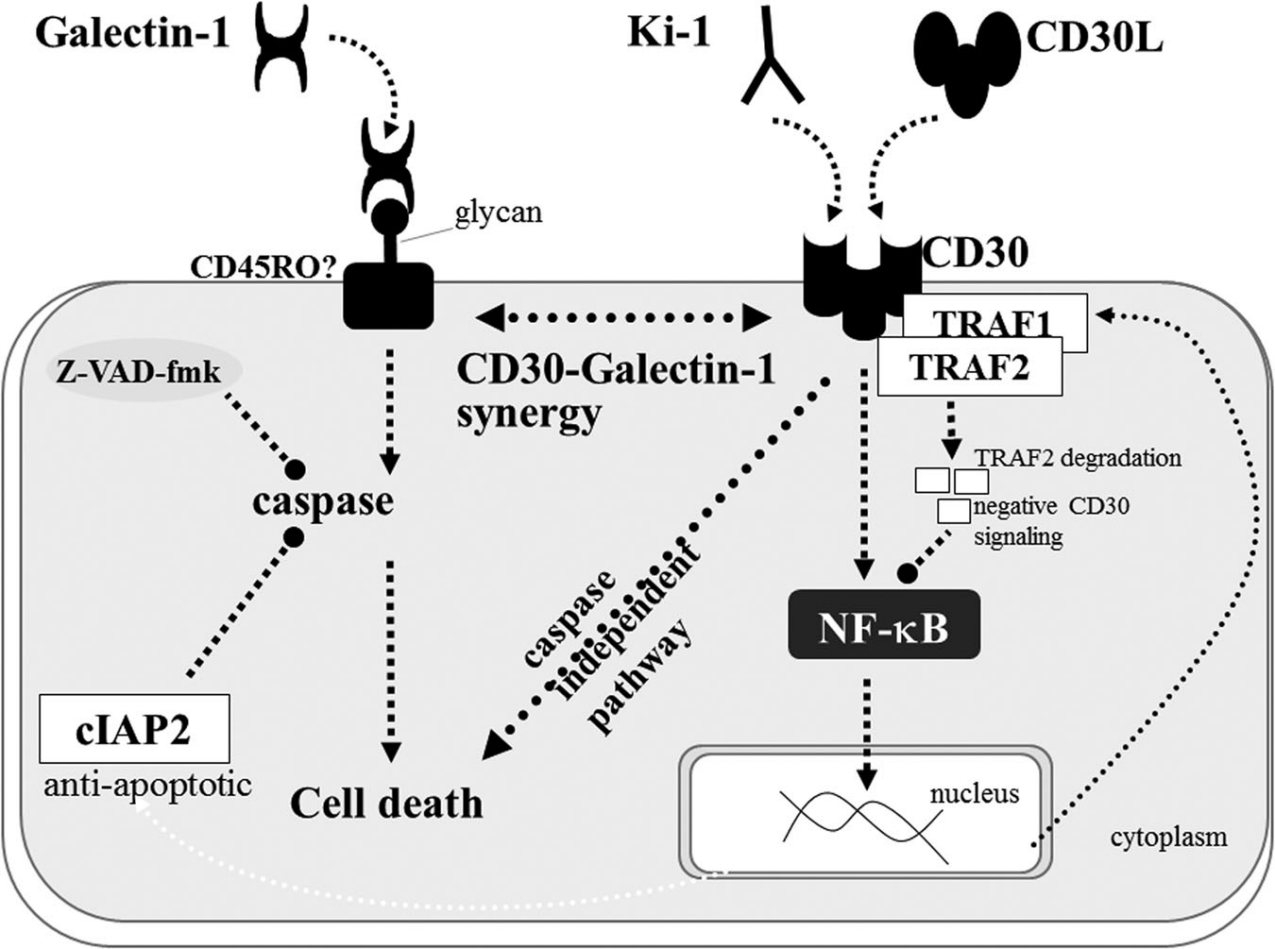
Schematic representation of CD30 signaling and galectin‐1 induced cell death. Synergistic effects may be induce enhanced cell death by galectin‐1 on prestimulation of CD30, but the detail synergistic mechanisms still remain unclear.

CD30 is a target for therapy of Hodgkin lymphoma.^33^, ^34^ We previously reported that CD30 prestimulation by the anti‐CD30 antibody Ki‐1 enhances galectin‐1 mediated lymphoma cell apoptosis in the ALCL cell line Karpas 299.^35^ Western blot analysis showed that anti‐CD30 antibody stimulates upregulation of tumor necrosis factor receptor associated factor (TRAF) 1 and (inhibitor of apoptosis protein‐2) cIAP2, and downregulation of TRAF2 in Karpas 299 cells. There is no difference in the expression patterns of TRAF1, TRAF2, and cIAP2 in Karpas 299 cells following prestimulation with or not stimulated with CD30 with respect to galectin‐1 induced apoptosis. The detailed mechanisms underlying the enhancement of galectin‐1 induced apoptosis by CD30 prestimulation remain unclear and there may be a synergistic effect between galectin‐1 and CD30 stimulation on the induction of apoptosis as shown in Fig. 7. This effect is not observed in all ALCL cell lines but may nonetheless be a candidate strategy for inducting apoptosis in ALCL, especially in light of recent research showing that CD30 is a therapeutic target for lymphoma,^36^ and CD30 is specifically expressed in Hodgkin or anaplastic large cell lymphoma. Taken together, galectin‐1 may be effective for treating anaplastic large cell lymphoma in combination with anti‐CD30 antibody. In other studies, complementary DNA plasmid expressing galectin‐3 was transfected into human embryonic kidney (HEK) 293 cells. Galectin‐3 was expressed on the cell surface and the cells exhibited marked aggregation. Galectin‐3 was also expressed in ALCL cells and was secreted into the microenvironment in an autocrine fashion. Therefore, galectin‐3 may act as a cell to cell adhesion molecule, and this may be related to the cohesive cell growth which is a characteristic histological finding of ALCL lymphoma. In future we will clarify whether the biological behavior of ALCL cells is regulated by galectins, and whether galectins are useful tools for ALCL therapy by inducing apoptosis.

## FUTURE PERSPECTIVE

**Figure 8:**
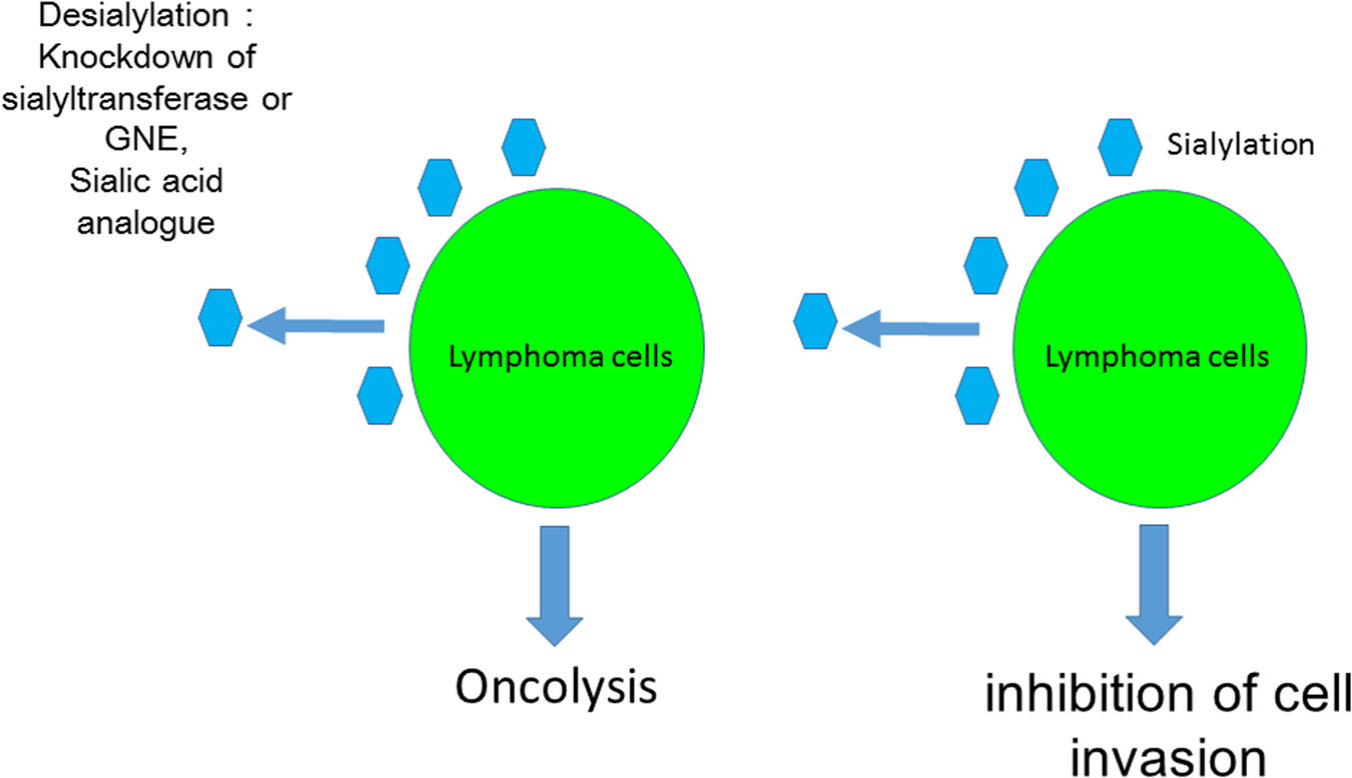
Desialylation of cell surface glycans as a strategy for lymphoma therapy. We previously demonstrated that sialylation is closely associated with the aggressiveness of lymphoma cells. Therefore, desialylation may inhibit lymphoma cell invasion and enhance oncolysis. Desialylation by knockdown of sialyltransferase or UDP‐GlcNAc 2‐epimerase (GNE), or inhibition of sialyltransferase by sialic acid analogues may provide new strategies for lymphoma therapy.

We have previously reported many biological phenomena related to the glycobiology of human lymphoma. Modulation of cell surface glycosylation by enzymatic or genetic methods may provide useful strategies for controlling lymphoma cells. However, more detailed understanding of the underlying mechanisms are required to induce effective killing of lymphoma cells by altering their glycosylation. We have already clarified the significance of glycosylation in understanding lymphoma cell biology and believe that modulating the sialylation of a cell surface is the most promising candidate strategy for altering lymphoma cell behavior, given that desialylation of the cell surface by neuraminidase treatment, or knockdown of ST6Gal1 or GNE, may lead to cell death induced by Fas or anticancer drugs. In addition, the inhibition of sialyltransferase induces the desiaylation of tumor cells.^37^ For example, a fluorinated sialic acid analogue is reported to lead to desialylation in tumor cells. A previous report suggested that a sialic acid analogue alters the cell surface sialylation status.^38^ The fluorinated sialic acid analogue 3Fax‐peracetyl Neu5Ac inhibits sialyltransferase and reduces cell surface sialylation in the Ramos B cell line.^39^ In the B16 melanoma cell line, this fluorinated sialic acid analogue inhibits sialylation and cell adhesion, migration, and growth.^40^ Sialic acid analogue complementation may allow artificial modification of cell surface sialylation and modulation of cell adhesion, growth, and death. Glycoengineering using fluorinated sialic acid analogues such as 3Fax‐peracetyl Neu5Ac may provide a new strategy for desialylation and is closely associated with the inhibition of metastasis and the induction of cell death in lymphoma cell lines. In addition, soyasaponin 1 inhibits sialyltransferase^41^, ^42^ and thus may be a candidate for inhibiting sialyltransferase and modulating cell adhesion, migration, and death. These desialylation strategies may allow the inhibition of tumor cell invasion and metastasis, and induce cell death, as shown in Fig. 8. In conclusion, we will continue investigating potential glycotherapy strategies for lymphoma therapy based on altered glycosylation.

## DISCLOSURE STATEMENT

None to declare.
